# Specific Protein Antigen Delivery to Human Langerhans Cells in Intact Skin

**DOI:** 10.3389/fimmu.2021.732298

**Published:** 2021-10-21

**Authors:** Mareike Rentzsch, Robert Wawrzinek, Claudia Zelle-Rieser, Helen Strandt, Lydia Bellmann, Felix F. Fuchsberger, Jessica Schulze, Jil Busmann, Juliane Rademacher, Stephan Sigl, Barbara Del Frari, Patrizia Stoitzner, Christoph Rademacher

**Affiliations:** ^1^ Department of Biomolecular Systems, Max-Planck-Institute of Colloids and Interfaces, Potsdam, Germany; ^2^ Department of Pharmaceutical Sciences, University of Vienna, Vienna, Austria; ^3^ Langerhans Cell Research Lab, Department of Dermatology, Venereology and Allergology, Medical University of Innsbruck, Innsbruck, Austria; ^4^ Department of Plastic, Reconstructive and Aesthetic Surgery, Medical University of Innsbruck, Innsbruck, Austria

**Keywords:** targeted delivery, C-type lectins, vaccine, subunit vaccine, transdermal, microinjection, Langerhans cells, Langerin

## Abstract

Immune modulating therapies and vaccines are in high demand, not least to the recent global spread of SARS-CoV2. To achieve efficient activation of the immune system, professional antigen presenting cells have proven to be key coordinators of such responses. Especially targeted approaches, actively directing antigens to specialized dendritic cells, promise to be more effective and accompanied by reduced payload due to less off-target effects. Although antibody and glycan-based targeting of receptors on dendritic cells have been employed, these are often expensive and time-consuming to manufacture or lack sufficient specificity. Thus, we applied a small-molecule ligand that specifically binds Langerin, a hallmark receptor on Langerhans cells, conjugated to a model protein antigen. *Via* microneedle injection, this construct was intradermally administered into intact human skin explants, selectively loading Langerhans cells in the epidermis. The ligand-mediated cellular uptake outpaces protein degradation resulting in intact antigen delivery. Due to the pivotal role of Langerhans cells in induction of immune responses, this approach of antigen-targeting of tissue-resident immune cells offers a novel way to deliver highly effective vaccines with minimally invasive administration.

## 1 Introduction

In early 2020, the Severe Acute Respiratory Syndrome Coronavirus 2 (SARS-CoV2) exposed humanity’s social and economic vulnerability towards unknown viruses. The spontaneous spread of zoonotic diseases (*i.e.* Influenza, Ebola, West Nile virus, SARS/MERS) is becoming a threat to our mobile and globalized world. Hence, novel efficient and rapid vaccine developments are required. Advanced approaches are explored, such as cell-culture-based virus manufacturing for instance. However, this suffers from high production costs. Other attempts emerge that aim to harness the immune system to fight pathogens with mRNA- and adenoviral vector-based vaccines. Using information from genetic sequencing, these technologies can develop nucleic acids encoding for antigens in short time.

A more direct way to generate immunity is by administration of a recombinant protein antigen as a subunit vaccine. This technology has some clear advantages and subunit vaccines against human papilloma virus and SARS CoV2 have been already marketed (1, 2). The absence of a carrier suggests lower hurdles for regulatory approval. However, rapid clearing of small proteins and low stability of the proteins in general usually require large amounts of administered material; therefore increasing production costs. To overcome these limitations, antigens can be targeted to immune cells as has been described by covalently binding to antibodies and glycan structures (3–9). Following this idea, we aim to address the above shortcomings by i) a novel targeted delivery mediated by a highly specific ligand to specialized immune cells (i.e. active targeting) in combination with ii) tissue-specific administration where those cells reside (i.e. passive targeting).

The targeted cells are Langerhans cells (LCs), predominantly found in the uppermost layer of the skin – the epidermis. Here, they fulfil the task of pathogen recognition, followed by antigen processing and elicitation of immune responses in the skin-draining lymph nodes. LCs promote an efficient T cell response as well as humoral immunity by B cell activation and expansion of T follicular helper cells – both crucial hallmarks for an effective vaccine (10–14). Their remarkable sensitivity to activate the cells of the adaptive immune system has been reported (15).

The high density of LCs renders the skin a promising vaccination access point, especially with their higher immune cell density compared to tissues reached *via* intramuscular or subcutaneous injection (16). Choosing the skin as an administration site for targeted delivery of vaccines holds the promise of lowering the amount of required protein vaccine. It further offers the opportunity to employ minimally invasive administration routes reducing the risk of injury and increasing patient compliance. Rouphael et al. recently demonstrated the induction of an efficient systemic immune response by an influenza vaccine administered *via* microneedle patches (17).

A unique cell surface marker allowing for targeted delivery to LCs is Langerin. This pathogen recognition receptor is a C-type lectin that recognizes mannose, *N*-acetyl-glucosamine and fucose structures on viruses, bacteria and fungi as well as self-antigens (18, 19). This receptor facilitates antigen uptake and transport into endosomal compartments, where antigens are released due to an acidic environment. This endosomal antigen release allows the receptor to recycle back to the cell surface for repeated engulfment of antigens - a process associated with cross-presentation to induce a CD8^+^ as well as a strong CD4^+^ T cell response (20). High-affinity antibodies do not necessarily follow the same pathway and Langerin-mediated antigen uptake might be a more favorable alternative. In fact, since the strong binding of antibodies may prevent endosomal release from the Langerin receptor, as it has been shown for other recycling receptors (21), accumulation of antigen by repeating “uptake-internalization-release-surface recycling”- cycles is reduced. This accumulation, however, is associated with an increase of exogenous antigen uptake and may lead to improved cross-priming of CD8+ cells (22). This is achieved by Ca2+ dependent coordination of our targeting ligand to Langerin, which enables pH dependent release in endosomal compartments and cargo accumulation upon receptor cycling (23).

We recently reported on the first small molecule-based ligand that is highly specific for Langerin (24). This ligand can be applied to functionalize nanoparticles which encapsulate drugs, antigens or toxins to be delivered into LCs (24, 25). The underlying recognition process depends on a multivalent recognition of the glycomimetic ligand mimicking the glycocalyx of a pathogen. Noteworthy, no LC activation was observed after targeted delivery of empty Langerin-ligand targeted liposomes *in vitro* (24).

Based on the above, we reasoned that directly functionalizing protein antigens with our Langerin-specific targeting ligand would expedite uptake by LCs to an extent that will outcompete the processes of protein degradation. This approach of targeted antigen delivery would significantly reduce a) regulatory complexity, b) manufacturing effort and c) production costs. In our study we used Green Fluorescent Protein (GFP) as a model protein antigen, conjugated it with multiple copies of the LC-specific targeting ligand and investigated binding and uptake processes *in vitro* (model cell lines and primary LCs) as well as *ex vivo* in human skin explants. We explored two administration routes, *i.e.* conventional intradermal injection and NanoPass microneedles. This work demonstrates the successful and specific uptake of targeted GFP (t-GFP) by LCs, rendering direct antigen targeting a promising prospect for next-generation immunotherapies and vaccines for efficient activation of the immune system.

## 2 Results

**Figure 1:**
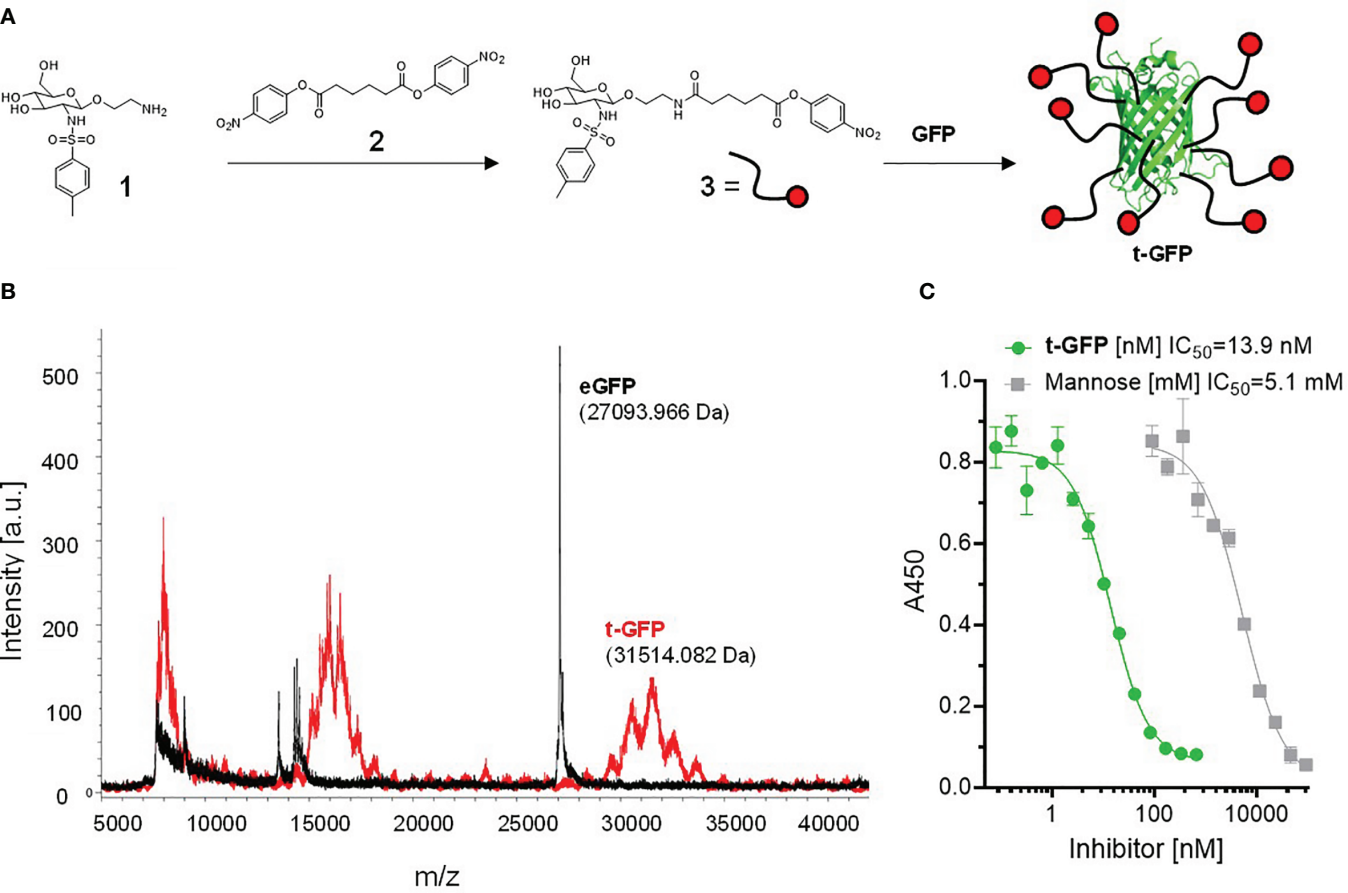
Conjugation of Langerin targeting ligand to GFP: **(A)** preparation of active ester 3 and subsequent reaction to t-GFP. **(B)** Determination of coupling efficiency by MALDI-TOF. The average m/z difference of GFP and t-GFP was divided by the molecular weight of the active ester 3 (minus the PNP leaving group). **(C)** ELLA showing inhibition curves of recombinant human Langerin binding mannan and t-GFP, graph shows n = 3 average ± SD.

To directly conjugate proteins with the Langerin targeting ligand 1, we synthesized the bis(4-nitrophenyl)adipate (PNP)-activated ester 3 which was subsequently coupled to lysins of GFP under mild aqueous conditions (**Figure 1A**) (26). A coupling efficiency of 9 to 10 ligands/protein (out of a total of 21 lysins) on the resulting t-GFP was determined by MALDI-TOF and ^1^H-NMR (**Figure 1B** and **Figure S1**). The clear and distinct signals obtained from the NMR experiment also confirm the maintained flexibility of the ligand and its solvent exposure.

To evaluate whether t-GFP binds to its target receptor, we used an enzyme-linked lectin assay (ELLA) in which binding of the extracellular domain (ECD) of Langerin to immobilized mannan can be inhibited; with an IC50 of 13.9 nM compared to 5.1 mM for mannose, we consider t-GFP to be a potent inhibitor of mannan-Langerin binding (**Figure 1C**).

**Figure 2:**
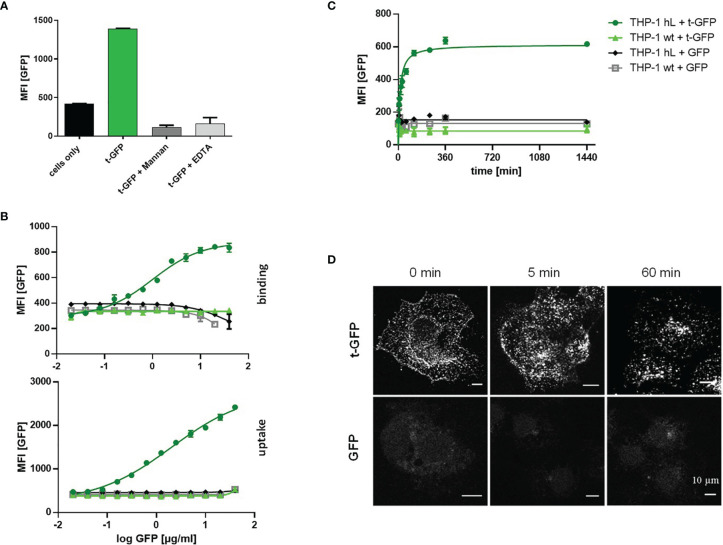
t-GFP shows specific binding and uptake by Langerin-expressing cell lines. **(A)** t-GFP binds to Langerin+ THP-1 cells and is inhibited by mannan and EDTA. n = 3 **(B)** binding and uptake of t-GFP occurs in a dose-dependent manner **(C)** kinetic analysis of t-GFP uptake into THP-1 cells. n = 3 **(D)** Fluorescence microscopy of Langerin^+^ COS7 cells shows rapid and selective uptake of t-GFP in comparison to non-targeted GFP.

Since C-type lectins on immune cells recognize various glycans, cell subset specific targeting using carbohydrates as targeting ligands is inherently difficult. The ligand employed in this work has previously been shown to selectively interact with Langerin, disregarding other related receptors such as DC-SIGN or Dectin-1/2 (24). This specificity was demonstrated for liposomes bearing hundreds of ligand entities on their outer lipid membrane providing multivalent binding to the receptor. To validate that also much smaller proteins (with fewer ligand copies attached) are able to mediate recognition and subsequent uptake by Langerin, we used cell lines (THP-1 and Raji) expressing the human Langerin receptor (hL) and wildtype cells (wt) as a control and exposed them to t-GFP (**Figure 2**). Indeed, only Langerin^+^ cells were able to bind t-GFP, whereas DC-SIGN^+^ or wt cells were not (**Figure 2A**). Non-targeted GFP did not interact with any of the cell lines tested, confirming protein uptake is solely mediated by the targeting ligand and its receptor. Binding of t-GFP to Langerin^+^ cells can be inhibited in the presence of EDTA or competitive amounts of mannan, a mannose polymer with high affinity for Langerin, which proves the ligand-receptor interaction to take place at the Ca^2+^-dependent carbohydrate recognition domain of Langerin (**Figure 2A**). Analysing dose-response studies, binding was saturated at concentrations above 10 µg/mL and uptake capacities were expectedly higher (**Figure 2B**). Kinetic studies of t-GFP uptake confirm a fast onset within minutes of incubation with a transit to saturation after 2 h and t_1/2 =_ 44_ min_ and no observed degradation after 24 hours (**Figure 2C**). Confocal microscopy of Langerin-expressing COS-7 cells additionally illustrates the rapid uptake of t-GFP and subsequent accumulation in endosomal compartments (**Figure 2D**). In comparison, GFP did not bind to any of the cells investigated.

**Figure 3:**
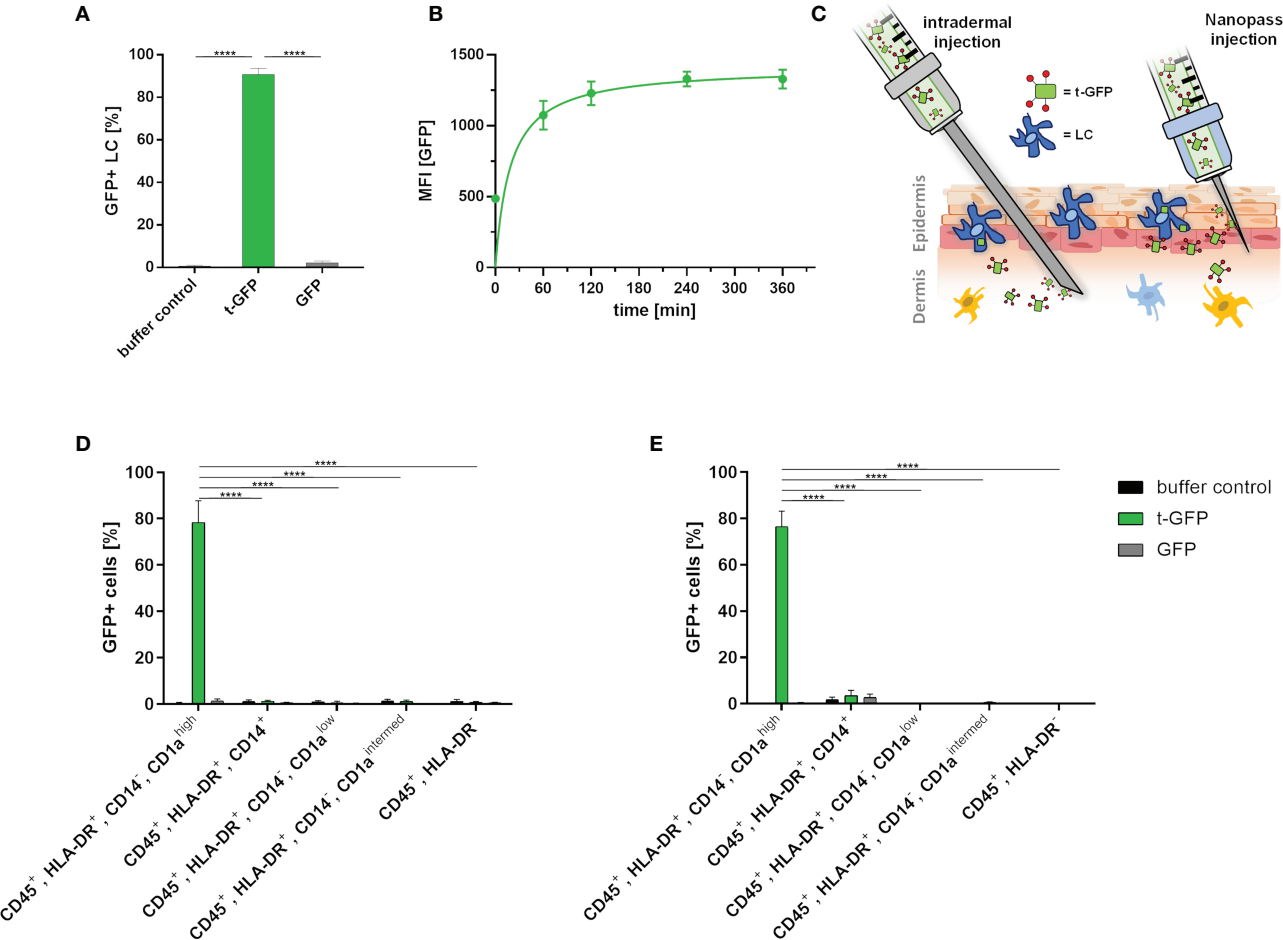
t-GFP is specifically taken up by LCs *ex vivo*. **(A)** flow cytometry analysis of t-GFP uptake by LCs in epidermal cell suspensions. n = 4 **(B)** kinetic analysis of t-GFP uptake into LCs purified *via* CD1a-microbeads. **(C)** schematic illustration of different administration routes for LC targeting in intact human skin. **(D, E)** specific uptake of t-GFP into LCs upon **(D)** intradermal (n = 3) and **(E)** NanoPass injection (n = 3). LCs were identified as **(A)** single, live, CD45^+^, HLA-DR^+^, Langerin^+^ **(D, E)** single, live, CD45^+^, HLA-DR^+^, CD14^-^, CD1a^high^. ****p-value < 0.0001, two-tailed, unpaired t-test.

Next, we applied the same approach to LCs which were obtained by enzymatic digestion of human skin (**Figure 3**) (27). Upon incubation of epidermal cell suspensions with t-GFP, 99% of LCs were t-GFP-positive after 1 h and 25 µM t-GFP concentration (**Figure 3A** and **Figure S2**). In contrast, GFP did not bind to LCs nor was it taken up. Similar to cell lines, uptake by LCs occurs within minutes and is saturated after 1-2 h (**Figure 3B**) without observable degradation, thereby suggesting no rapid enzymatic digestion of the protein antigen in LCs in line with previous reports (28).

To translate the above *in vitro* results from cell culture into a more realistic tissue context, we explored two different intradermal administration routes on *ex vivo* human skin explants (**Figure 3C**). It is important to stress that the identification of the best administration route has to take several parameters into account: manufacturing costs, up-scalability, patient compliance, and off-target-tissue distribution. Conventional intradermal injections are not only the most common and cheapest way to deliver antigens to DCs, but are also approved by regulatory agencies. Drawbacks, however, are risk of needle-caused injuries, necessity of trained personnel, and low patient compliance. Hence, we explored the delivery of t-GFP by i) conventional needles (BBraun Sterican^®^ 30Gx1/2’’) and ii) NanoPass^®^600 injection devices - disposable syringe tips bearing three slanted 600 µm needles for solution injection. The latter technology is FDA-approved and, after minimal training, provides a safe and painless administration for the patients (29). NanoPass silicon crystal needles are robust enough to be used multiple times for one injection site (**Figure S3**). This allows for several low volume injections reducing the pressure a single prick would create. We consider this advantageous as it also increases the treated area and therefore the number of LCs. It also simultaneously decreases the probability of antigen solution being pushed down into subcutaneous tissue by large injection volumes. For this comparison, skin explants were either digested to generate epidermal cell suspensions (**Figure S3**) or cultured on medium to allow skin DCs to emigrate from skin explants (27). This allows for analysis of those cell populations that migrated out of the explants, thereby mimicking the *in vivo* activation process of LCs (**Figures 3D, E**).

Despite the intradermal injection of t-GFP, 78.4% of LCs that emigrated from the epidermis were positive for t-GFP, whereas a mere 1.2% of dermal monocyte-derived macrophages (CD45^+^, HLA-DR^+^, CD14^+^); 0.6% and 1.0% of dermal DCs (CD45^+^, HLA-DR^+^, CD14^-^, CD1a^low^ and CD45^+^, HLA-DR^+^, CD14^-^, CD1a^intermed^, respectively) and 0.8% of other leukocytes (CD45^+^, HLA-DR^-^) were positive for t-GFP. These low amounts of off-target cells once more emphasized the high specificity of our targeting Langerin-ligand for LCs in clear distinction to various CLRs like DC-SIGN, Dectin-1/2, Mannose-receptor and DEC205 - all present on dermal DCs in crucial spatial proximity to LCs *in vivo*. GFP on the other hand showed little to no binding or uptake by any other cell type (**Figure 3D** and **Figure S4**).

Using NanoPass injection devices, 76.6% of the LCs that have migrated out of the explants were t-GFP^+^, without any binding or uptake of GFP (**Figure 3E** and **Figure S4**). 3.5% of dermal monocyte-derived macrophages (CD45^+^, HLA-DR^+^, CD14^+^); 0.0% and 0.5% of dermal DCs (CD45^+^, HLA-DR^+^, CD14^-^, CD1a^low^ and CD45^+^, HLA-DR^+^, CD14^-^, CD1a^intermed^, respectively) and 0.1% of other leukocytes (CD45^+^, HLA-DR^-^) were positive for t-GFP. Epidermal cell suspensions generated from injected skin areas likewise obtained 98% t-GFP+ LCs at 10 µg/ml injected concentration (**Figure S3B**). Furthermore, to study LC-specific priming of both T and B cells, the latter requires presentation of intact antigen for germinal center formation in local lymph nodes (30, 31). With cells harvested after 4- 5 days of skin explant culture (**Figures 3D, E**) our data indicate that protein antigens incorporated by ligand-mediated uptake are not enzymatically digested in the endo-lysosomal compartments as GFP is still functional in emigrated LCs. This time frame would allow delivery of intact protein antigen by LCs to B cells in draining lymph nodes. The presence of antigen in emigrated LCs after intradermal injection and NanoPass application strongly suggests that our novel Langerin-mediated delivery system enables LCs to transport protein antigen to the draining lymph nodes for subsequent antigen presentation.

## 3 Discussion

LCs are capable to present and cross-present antigens leading to both cytotoxic T cell responses and germinal center formation triggering antibody production, which makes them a promising target for immunotherapeutic approaches. Within this study we show the first steps of this cascade towards intradermal, subunit vaccination by specific delivery of protein antigen.

Based on protein conjugation strategies developed in the last years conjugation of targeting ligands to proteins became a feasible and well established method that aids vaccine improvement (26). Using a glycomimetic targeting ligand, we aim to imitate natural glycan recognition of skin-invading pathogens by the Langerin receptor, which promotes pathogen clearance and immunoprotection. Highlighting the relevance of the targeting ligand, binding or uptake of non-glycosylated protein was not observed (**Figures 2C** and **3A**). Although successful for DC-SIGN, Langerin targeting with natural ligands such as mannose has been shown insufficient (32, 33). From this we conclude a certain affinity/avidity threshold that needs to be overcome to facilitate Langerin-targeted delivery. By direct protein conjugation to a glycomimetic ligand and intradermal administration into intact human skin, we surpassed this threshold and achieved cell-specific targeting of protein antigens to LCs.

With LC targeting efficiencies of 80-100%, at just ~1% off-target cells (including dermal DC subsets, see **Figure 3D**), these results indicate a substantial efficiency increase compared to non-targeted approaches. In a vaccine context, this promises reduction of required treatment dosage, translating to i) faster vaccine distribution within a population and ii) presumably reduced side effects - both combined with the benefits of minimally invasive dermal application.

LCs that emigrated from the epidermis were positive for GFP protein antigen, which implies the protein was not rapidly degraded. Hence, we suspect intact antigen will also be efficiently transported to and presented in the draining lymph node *in vivo*. In line with previous findings on protein persistence in LCs and T cell priming (28), our results support the view, that it is not passive diffusion *via* the lymph, but active transport by LCs that delivers protein antigen to local lymph nodes. Accordingly, future focus lies on confirmation of our findings *in vivo*, shedding light on downstream aspects of this cascade, from antigen processing and presentation to effector cell activation.

## 4 Methods

### 4.1 GFP Recombinant Production

Log Phase *E. coli* BL21 bearing plasmid pRSET His-eGFP (addgene #113551, a kind gift by from Jeanne Stachowiak) were cultured overnight expressing the protein. Bacteria were then pelleted, washed, and sonicated on ice to extract the protein. Protein was purified using a Ni-NTA column (Thermo Fisher) according to the manufacturer’s specification.

### 4.2 Preparation of t-GFP

#### 4.2.1 Linker Conjugation

The human Langerin targeting ligand [14 mg, described by Wamhoff et al. (24)] was dissolved in 1900 µL dimethyl sulfoxide (DMSO, SIGMA 276855-100mL, LOT# STBH9909). 784 µL pyridine (Merck 1.09728.100, LOT# K44424528320) and 325 µL triethylamine (SIGMA T0886-100mL, LOT# STBD4118V) were added. Bis(4-nitrophenyl)adipate (PNP)-activated ester **3** (157 mg) was dissolved in 1700 µL DMSO and the solution was added to the solution containing the targeting ligand. After 3 h stirring, the stirring bar was removed, and the reaction mixture lyophilized overnight.

#### 4.2.2 Ligand-Linker Purification

The residue was washed with cold (4°C) toluene (4x 5 mL). TLC was used to check that all excess linker was washed away and no ligand dissolved (TLC 60:40 Hex : EtOAc). Solvent was removed *in vacuo* and the residue dried in high vacuum for 16 h.

#### 4.2.3 GFP Conjugation

12.7 mg GFP (see above) in 6 mL PBS (pH 8) were added to the flask containing the activated ligand-linker-construct from step 2. A stirring bar was added, and the solution slowly stirred for 17h at room temperature.

#### 4.2.4 Targeted GFP (t-GFP) Purification

2 x 3 mL of the mixture were diluted to 12 mL (with PBS pH 7.4), and centrifuged to remove precipitated protein (60 min, 15000 RCF). The supernatant was transferred into a dialysis cassette (Thermo Scientific 66710, LOT# P00001531) and dialyzed over night at 4°C against PBS pH 7.4 (2x 2L).

#### 4.2.5 Concentration of t-GFP

The solution from step 4 was concentrated to 2.8 mg/mL by a 15 mL Amicon (Merck UFC901024, LOT# R9NA92300) at 7000g (1x5 min, 1x 3 min), syringe filtered (Corning 0.45 um 431220, LOT# 11519021) and stored at -20°C.

#### 4.2.6 Quantification of Coupling Efficiency by MALDI-TOF

A sample taken out of the above storage solution was desalted prior to analysis:

A Zip Tip C4 (Merck) was rinsed with 10 µL H_2_O (+0.1% trifluoroacetic acid), then H_2_O, then H_2_O/acetonitrile (3:2) and pushed dry. 10 µL of protein sample was loaded into the Zip Tip and washed with 10 µL H_2_O. The sample was eluded with 10 µL H_2_O/acetonitrile (3:2) directly onto the matrix. The sample was analysed by MALDI-TOF-MS (MALDI-TOF Autoflex Speed MS Bruker Daltonics Bremen, Germany) using 2′,6′-Dihydroxyacetophenone (DHAP) as matrix and a reflector method in positive ion mode.

The coupling efficiency of 9 ligands/protein was calculated by subtracting the mass of un-targeted GFP (27,094 Da) from the mass measured for t-GFP (31,500 Da) followed by division of the molecular weight of the ligand-linker construct (excluding the *p*-nitrophenyl leaving groups= 488 g/mol).

#### 4.2.7 Quantification of Coupling Efficiency by NMR

The t-GFP concentration was determined *via* Nanodrop (extinction coefficient of GFP at 280 nm: 0.94) Note: The ligand does not absorb at 280 nm interfering with the measurement.

The NMR sample of t-GFP NMR was prepared in a 3 mm NMR tube (Norell Select Series 3 mm HT) as follows (DSS and D_2_O were obtained from Sigma Aldrich):

- 10 µM final concentration of functionalized protein (in PBS, pH 7.4)
- 10 µM final concentration 3-(Trimethylsilyl)-1-propanesulfonic acid-d6
- 15 µL (final concentration 15%) D_2_O
- MQ water was added to top up the mixture to 150 µL

The NMR experiment was conducted on a Bruker Ascent 700 using a water suppression pulse programme accounting for 10% D_2_O in the sample.

The coupling efficiency of the targeting ligand to GFP was determined as followed using MestreNova (Mestrelab):

Without any baseline correction, the integrals of the DSS signal was set to 9 protons and the integrals for the aromatic signals of the ligand were determined. Each of the two signals represent two protons of the ligand; hence the integral was divided by 2 yielding a coupling efficiency of 10 ligands/protein.

### 4.3 Mannan ELLA

An enzyme-linked lectin assay (ELLA) using mannan coated 96 well maxisorb plates (Nalge Nunc International) has been described by Aretz et al. (34) In short, plates were coated with a mannan solution overnight and blocked with a BSA solution. After washing, a solution of Langerin ECD at 5 nM in 100μL HBS-P (25 mM HEPES, pH 7.6, 150 mM sodium chloride) with 2 mM calcium chloride was added to the plates and incubated at room temperature for 2h. This incubation was done in the presence of different concentrations of either t-GFP or mannose as inhibitors. After washing, plates were incubated with Streptactin-HRP conjugate, washed again, and developed with TMB solution (Rockland, Gilbertsville, PA, USA). The reaction was stopped with 0.18 M sulfuric acid and absorption was measured at 450 nm. Data points were fitted using an inhibitor *vs.* response function (software: GraphPad Prism 8).

### 4.4 Cell Culture

If not stated otherwise, all media and supplements were purchased from Thermo Fisher Scientific. The human THP-1 cell line from monocytic origin was grown in full growth medium of RPMI1640 medium supplemented with 10% FCS, 50 µg/ml Gentamycin and GlutaMax. Cells were maintained between 0.5 and 1 Mio cells/ml by addition or replacement of complete growth medium. Cells were cultured in 10 cm or 15 cm petri dishes (greiner) in 10 ml or 20 ml culture medium, respectively. Cells were monitored with a light microscope (IT40 5PH, VWR) and grown under controlled conditions at 37°C and 5% CO_2_. For cell splitting, cells were collected and centrifuged at 500g for 3 min (Heraeus Megafuge 8R, Thermo Fisher Scientific). The supernatant was aspirated and cells were resuspended in fresh growth medium. Cell lines expressing Langerin were generated as described before^1^. Briefly, human Langerin cDNAs (Sinobiologicals) were cloned into a lentiviral BIC-PGK-Zeo-T2a-mAmetrine : EF1A construct by Gibson assembly (NEB) according to the manufacturer’s protocol. Hek293 cells were transfected S4 with the lentiviral vector together with third-generation packaging vectors and viral particles were then used for transduction of THP-1 cell lines.

### 4.5 Kinetic Analysis of t-GFP Uptake Into THP-1 Model Cells

50.000 cells per well were plated in 100 µl full growth medium supplemented with 10 µg/ml GFP or t-GFP respectively. Samples were incubated at 37°C for indicated time-points from 0-24h. Following incubation, samples were placed on ice and washed with 200 µl ice-cold HBSS supplemented with 10 mM EDTA to remove surface-bound GFP. Finally, cells were resuspended in 200 µl HBSS and GFP fluorescence was detected *via* flow cytometry (Attune Nxt, life technologies).

### 4.6 Dose-Response Analysis of t-GFP Binding and Uptake to THP-1 Model Cells

50.000 cells per well were plated in 45 µl full growth medium in 96-well U-bottom plates (greiner) on ice. 1:2 serial dilutions of GFP and t-GFP were prepared in DPBS and added in 5 µl to obtain indicated final concentrations from 0-40 µg/ml. After incubation for 1 h on ice (for binding assays) or 37°C (for uptake assays), cells were washed with 200 µl ice-cold HBSS. For uptake assays, cells were washed with 200 µl ice-cold HBSS supplemented with 10 mM EDTA to remove surface-bound GFP. Finally, cells were resuspended in 200 µl HBSS and GFP fluorescence was detected *via* flow cytometry (Attune Nxt, life technologies).

### 4.7 Fluorescence Microscopy of Langerin+ COS-7 Cells

hLangerin+ COS-7 cells were seeded on non-coated glass coverslips in a 12-well tissue culture dish (Sigma Aldrich) in 1 ml DMEM supplemented with 10% FCS, 100 IU/ml penicillin and 100 μg/ml streptomycin. The next day, cells were incubated with t-GFP or GFP at a final concentration of 5 µg/100µl for 1h at 4°C in ice cold medium. For t=0, the initial time point, cells were washed with ice cold PBS with 2 mM magnesium chloride and 2 mM calcium chloride (invitrogen) at 4°C and fixed in 4% Roti-Histofix (Roth) for 10 min at RT. For the remaining time points, cells were washed once in PBS with 2 mM magnesium chloride and 2 mM calcium chloride, incubated for 5 or 60 min at 37°C in warm complete DMEM, washed with warm PBS with 2 mM magnesium chloride and 2 mM calcium chloride and subsequently fixed. The localization of GFP was visualized with an SP8 confocal microscope (Leica).

For the following experiments, healthy human skin samples were collected after informed consent and approval by the local ethics committees AN5003 323/4.10 403/5.10 (4470a) and MPG2018_16:

### 4.8 GFP NanoPass Injection and Skin Sample Preparation

Skin samples from abdominal reductive reconstruction were placed on Styrofoam plates and using NanoPass^®^600 microneedle devices several low volume injections of 20-50 µl were made up to 500 µl total injection volume. Herein, GFP and t-GFP was applied at 10 µg/ml in DPBS. After 1h incubation at RT skin grafts were prepared with an electric dermatome (Zimmer) at 0.4 mm thickness and incubated in DPBS supplemented with 10 µg/ml gentamycin for 30 min.

### 4.9 Generation of Epidermal Cell Suspensions

For preparation of epidermal cell suspension, injected skin areas (for NanoPass experiments) or untreated skin grafts (for uptake experiments with epidermal cell suspensions) were incubated with the epidermis floating upwards in RPMI1640 supplemented with 1.5 U/ml dispase II (Roche) and 0.1% trypsin (sigma) overnight at 4°C. Next morning after an additional incubation for 1h at 37°C, the epidermis was peeled of using tweezers, transferred into complete growth medium, minced into smaller pieces and finally filtered through a 100 µm and a second 40 µm cell strainer to obtain single cell suspensions.

### 4.10 GFP Uptake by LC in Epidermal Cell Suspensions

50 µg/ml GFP or t-GFP were incubated with epidermal cell suspensions in full growth medium for 1 h at 37°C and uptake was measured as **GFP**-positive cells by flow cytometry.

### 4.11 Skin Migration Assay

#### 4.11.1 NanoPass Injections

8 mm punch biopsies (Kai Medical) were prepared from injected and grafted skin sections. Punches were placed in 100 µm cell strainers floating on 6 ml full growth medium in 6-well plates and incubated at 37°C. Half of the culture medium was carefully replaced after 2d. On day 4 the supernatant was harvested, wells were rinsed with ice-cold DPBS 10 mM EDTA, and samples were further processed for flow cytometric analysis.

#### 4.11.2 Conventional Needle Injection

1 μg of **GFP** in 25 μl HBSS were injected i.d. into 8 mm skin biopsies with a 30G syringe. Skin explants were placed onto a 100 μm cell strainer in 6 ml full growth medium in a 6-well plate. After 4 days, cells were harvested and analyzed for GFP signal by flow cytometry.

### 4.12 Flow Cytometry Analysis of Skin Suspension Samples

Dead cells were excluded by the fixable viability dye eFluor 780 (eBioscience) and FcR-dependent staining was blocked with human FcR blocking reagent (Miltenyi). Cells were stained with fluorophore-conjugated antibodies against CD45 (clone HI30, BioLegend), CD14 (clone HCD14, BioLegend), HLA-DR (clone L242, BioLegend), CD1a (clone HI149, BioLegend), human Langerin (clone MB22-9F5, Miltenyi) for either 30 min or 15 min on ice. Experiments were conducted with an Attune NxT flow cytometer (ThermoScientific) or FACS Canto II (BD Biosciences) and analysed in FlowJo software.

### 4.13 Enrichment of CD1a+ Epidermal Cells

Langerhans cells, identified as CD1a expressing epidermal cells, were enriched from epidermal cell suspensions by magnetic labeling with human anti-CD1a MicroBeads (Miltenyi) and subsequent positive-selection by running LS MACS columns (Miltenyi) twice according to the manufacturer’s instructions^1^.

### 4.14 Kinetic Analysis of t-GFP Uptake Into Primary LCs

CD1a-enriched epidermal cells were cultured in complete growth medium supplemented with 200 U/ml GM-CSF (Miltenyi) at 10.000 cells/well and t-GFP was added to 10 µg/ml in 100 µl sample volume. Samples were incubated at 37°C for indicated time-points from 0-6h. Following incubation, samples were placed on ice and washed with 200 µl ice-cold HBSS supplemented with 10 mM EDTA to remove surface-bound GFP. Finally, cells were resuspended in 200 µl HBSS and GFP fluorescence was detected *via* flow cytometry (Attune Nxt, life technologies).

## Data Availability Statement

The raw data supporting the conclusions of this article will be made available by the authors, without undue reservation.

## Author Contributions

MR: Methodology, Investigation, Formal analysis, Writing - Review & Editing, Visualization. RW: Methodology, Investigation, Formal analysis, Writing - Original Draft, Visualization. CZ-R: Investigation, Formal analysis. HS: Investigation, Formal analysis. LB: Investigation, Formal analysis. FF: Investigation, Formal analysis, Writing - Review & Editing, Visualization. JS: Methodology, Investigation, Formal analysis. JB: Investigation, Formal analysis. JR: Investigation, Formal analysis, Visualization. BF: Methodology. SS: Methodology. PS: Methodology, investigation, and manuscript editing. CR: Conceptualization, Supervision, Project administration, Funding acquisition, Writing - Review & Editing. All authors contributed to the article and approved the submitted version.

## Funding

The work was supported by grants DFG 1944/6-1 to C.R. and FWF-P33855 to PS.

## Conflict of Interest

The authors declare the following competing financial interest(s): RW, JS, and CR declare the filing of a patent covering the use of Langerin targeting ligands. RW and CR are shareholders of Cutanos GmbH.

The remaining authors declare that the research was conducted in the absence of any commercial or financial relationships that could be construed as a potential conflict of interest.

## Publisher’s Note

All claims expressed in this article are solely those of the authors and do not necessarily represent those of their affiliated organizations, or those of the publisher, the editors and the reviewers. Any product that may be evaluated in this article, or claim that may be made by its manufacturer, is not guaranteed or endorsed by the publisher.
