# Supplementary material for: Specific Protein Antigen Delivery to Human Langerhans Cells in Intact Skin
Source: Front Immunol. 2021 Oct 21;12:732298. doi: 10.3389/fimmu.2021.732298 (PMC8566742; doi:10.3389/fimmu.2021.732298)
Supplement: Supplementary file 1 [file DataSheet_1.docx]

Supplementary Material

# Supplementary Figures


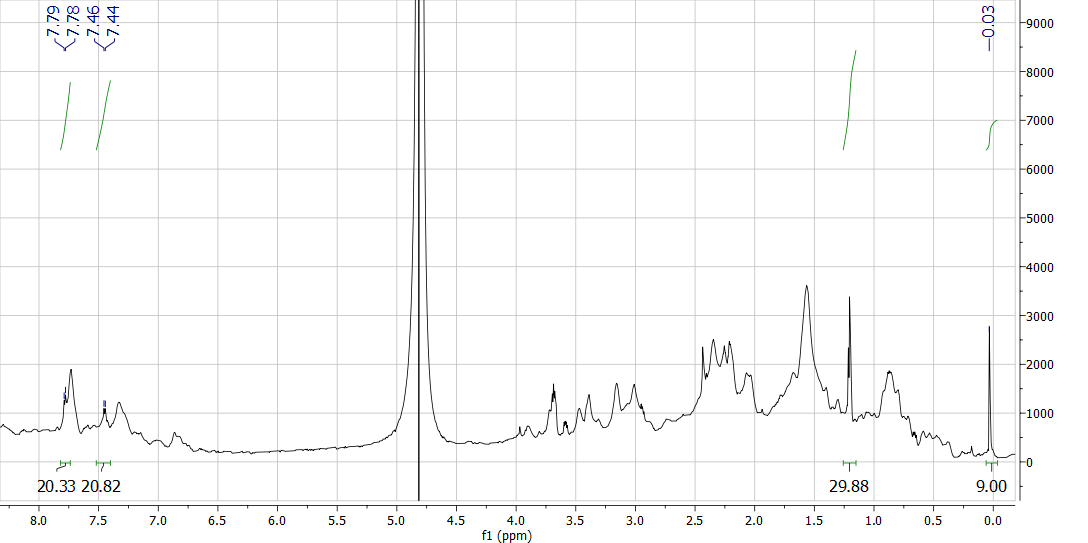


Supplementary Figure 1: Coupling efficiency determination by NMR by comparing the integrals of the ligand’s aromatic signals (7.4-7.8 ppm) to the integral of an internal standard (DSS, ~0 ppm). For experimental details see Methods section below.

**Supplementary** Figure 2: Analysis of epidermal cell suspensions generated from human skin explants. LCs were identified as single, live, CD45^+^, HLA-DR^+^, CD1a^+^, Langerin^+^ epidermal cells. **a)** targeted GFP, **b)** non-targeted GFP, **c)** buffer control.

**

**Supplementary** Figure 3: Analysis of epidermal cell suspensions generated from human skin explants after injection with NanoPass microneedle devices and incubation for 1h at RT. **a)** LCs were identified as single, live, Langerin^+^ epidermal cells. **b)** uptake of t-GFP by LCs compared to PBS buffer control. Pre-gating: single, live cells. **c)** Photography of NanoPass Micron Jet600 syringe tips with silicon crystal microneedles (600 µm in length).

**Supplementary** Figure 4: Characterization of emigrated skin DCs from skin explant cultures collected 4 days after injections of **a)** targeted GFP, **b)** non-targeted GFP and **c)** HBSS buffer via NanoPass or conventional intradermal needle injection. LCs were identified as single, live, CD45^+^, HLA-DR^+^, CD14^-^, CD1a^high^ cells.
